# Systematic Review and Meta-analysis of SurgicalTreatment of Non-Zenker’s Oesophageal Diverticula

**DOI:** 10.1007/s11605-017-3368-3

**Published:** 2017-01-20

**Authors:** David S. Y. Chan, Antonio Foliaki, Wyn G. Lewis, Geoffrey W. B. Clark, Guy R. J. C. Blackshaw

**Affiliations:** Department of Surgery, University Hospital of Wales, Heath Park, Cardiff, CF14 4XW UK

**Keywords:** Oesophageal diverticula, Myotomy

## Abstract

**Background:**

Oesophageal diverticula are rare outpouchings of the oesophagus which may be classified anatomically as pharyngeal (Zenker’s), mid-oesophageal and epiphrenic. While surgery is indicated for symptomatic patients, no consensus exists regarding the optimum technique for non-Zenker’s oesophageal diverticula. The aim of this study was to determine the outcome of surgery in patients with non-Zenker’s oesophageal diverticula.

**Methods:**

PubMed, MEDLINE and the Cochrane Library (January 1990 to January 2016) were searched for studies which reported outcomes of surgery in patients with non-Zenker’s oesophageal diverticula. Primary outcome measure was the rate of staple line leakage.

**Results:**

Twenty-five observational studies involving 511 patients (259 male, median age 62 years) with mid-oesophageal (*n* = 53) and epiphrenic oesophageal (*n* = 458) diverticula who had undergone surgery [thoracotomy (*n* = 252), laparoscopy (*n* = 204), thoracoscopy (*n* = 42), laparotomy (*n* = 5), combined laparoscopy and thoracoscopy (*n* = 8)] were analysed. Myotomy was performed in 437 patients (85.5%), and anti-reflux procedures were performed in 342 patients (69.5%). Overall pooled staple line leak rates were reported in 13.3% [95% c.i. (11.0–15.7), *p* < 0.001] and were less common after myotomy (12.4%) compared with no myotomy (26.1%, *p* = 0.002).

**Conclusions:**

No consensus exists regarding the surgical treatment of non-Zenker’s oesophageal diverticula, but staple line leakage is common and is reduced significantly by myotomy.

## Introduction

Oesophageal diverticula (OD) are rare outpouchings of the oesophagus with a prevalence of up to 3% based on radiologic and endoscopic studies.^1^^,^ 2 OD may be classified anatomically as pharyngeal (Zenker’s) which is the most common type (70%), middle and distal oesophageal (epiphrenic).^3^ The aetiology of non-Zenker’s OD can be divided into traction and pulsion. Traction diverticula are true diverticula (include all layers of the oesophagus) which are due to chronic mediastinal diseases.^4^ Pulsion diverticula are false diverticula (an outpouching of the mucosa or submucosa) caused by increased intraluminal pressure secondary to a motility disorder or mechanical obstruction.^5^^,^ 6

While surgery is indicated for symptomatic patients, no consensus exists regarding the optimum technique for non-Zenker’s OD (transabdominal versus transthoracic, open versus minimally invasive, diverticulectomy versus diverticulopexy, routine versus selective myotomy and the need for an anti-reflux procedure). This is because alterations in oesophageal motility are not simply detected despite great improvements in the understanding of the pathophysiology of oesophageal functional diseases.^5^^,^ 7 Although various disorders such as achalasia, hypertensive lower oesophageal sphincter and diffuse oesophageal spasm have been found to be associated with non-Zenker’s OD,^8^ histologic abnormalities of the oesophageal myenteric plexus were reported in 80% of patients in the absence of a specific motility disorder.^9^

Surgery is an effective treatment for non-Zenker’s OD but is associated with significant morbidity of up to 75% including staple line leak rates of up to 33% and mortality of up to 11%.^8^^,^ 10 ^–^ 13 These outcomes have not changed despite advancements in minimally invasive surgery and stapling devices.^14^^,^ 15 In the absence of randomised controlled trials, we conducted a systematic review and meta-analysis of observational studies to determine the optimal surgical approach in patients with non-Zenker’s OD.

## Materials and Methods

### Search Strategy

A systematic review of published work was conducted according to the Meta-Analysis of Observational Studies and Epidemiology (MOOSE)^16^ and Preferred Reporting Items for Systematic Reviews and Meta-Analyses (PRISMA)^17^ guidelines. A systematic search of PubMed, MEDLINE, EMBASE and the Cochrane Database of Systematic Reviews and Cochrane Controlled Trials Register, Cochrane Library, was performed by DC on March 1, 2016. A sensitive search strategy that combined the exploded thesaurus term for oesophageal diverticula or free text terms in the title or abstract for “oesophageal, epiphrenic diverticula” was developed. The searches were limited to human studies published in the English language from 1990 onwards. Further articles were identified by hand-searching reference lists of all articles retrieved to identify potentially relevant studies. Searches were cross-referenced on PubMed using the related articles function.

### Inclusion Criteria

Studies reporting surgical outcomes in patients with non-Zenker’s OD were included. When there were multiple articles by the same authors analysing data from the same or similar patient group, the most recent publication was included if the study periods overlapped.

### Exclusion Criteria

Studies of patients with pharyngeal (Zenker’s) diverticulum were excluded. Studies with less than five patients, review articles, case reports, nationwide databases based on coding, experimental studies and unpublished data from conference abstracts were excluded.

### Data Extraction

Data were extracted independently by the authors using a standard protocol. Any discrepancies were dealt with by discussion, and consensus was reached. The following information was extracted from each study: first author, year of publication, study design, country of origin, total number of patients, age, median follow-up, site and size of diverticula, presence of motility disorder, details of surgery, staple line leak, morbidity, mortality, reoperation, recurrence rates and presence of reflux symptoms at follow-up. Authors were not contacted for incomplete data. The primary outcome measure was the rate of staple line leakage. This was defined as a clinically relevant leakage over the diverticulectomy staple line which was confirmed radiologically. Secondary outcome measures include successful treatment (defined as symptom improvement or resolution at follow-up), morbidity, mortality, reoperation and recurrence rates and the presence of reflux symptoms at follow-up.

### Statistical Analysis

The meta-analysis was performed in line with the recommendations from the Cochrane Collaboration and PRISMA guidelines using Review Manager 5.3 (The Nordic Cochrane Centre, The Cochrane Collaboration, Copenhagen, Denmark).

Meta-analysis was used to pool study estimates of the outcome measures as detailed above. The pooled estimated outcomes were calculated using generic inverse variance random-effects meta-analysis using data from studies which reported at least one event in the outcome under investigation with standardised mean differences and 95% confidence intervals (c.i.) quoted. Patients who did not undergo diverticulectomy were excluded from calculations of staple line leak rates.

Subgroup analyses were conducted according to the surgical approach. Heterogeneity among study estimates was quantified using the *I* ^2^ value and associated test for heterogeneity which was reported for each analysis. Where heterogeneity was apparent, the DerSimonian and Laird random-effects method was used to pool estimates with inverse variance weights. The fixed-effects method of Mantel-Haenszel was applied otherwise.

### Study Quality

The quality of non-randomised studies was assessed using the Newcastle-Ottawa Scale which examines patient selection methods, comparability of study groups and assessment of outcome. A score of at least 6 stars from a maximum of 9 was considered to indicate higher quality.

## Results

### Characteristics of Included Studies

**Fig. 1:**
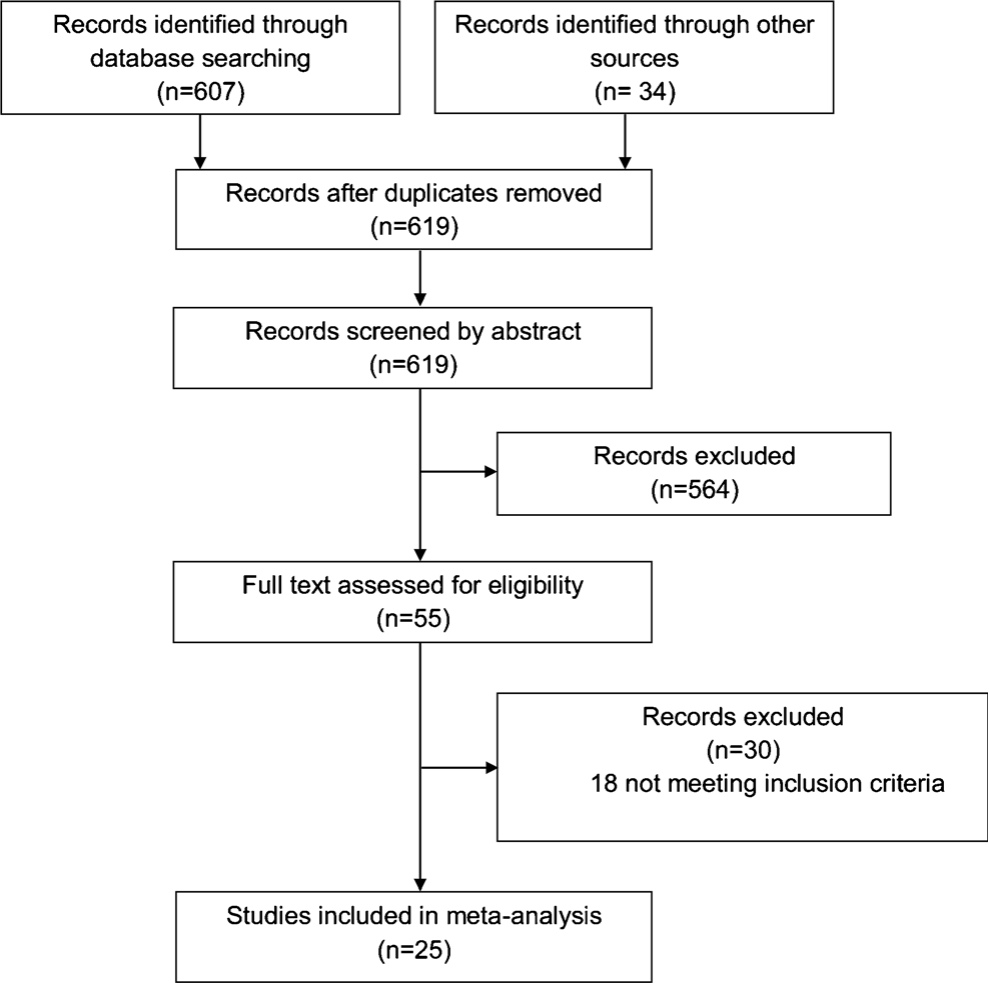
Identification process for eligible studies

The search identified 641 studies of which 25 were suitable for inclusion (Fig. 1). All studies analysed were observational cohorts, of which one had a prospective design^11^ (Table 1).

**Table 1:** Characteristics of included studies

Author	Year	Country	Total	Age (years)	Approach	Myotomy	Anti-reflux	F/U^a^ (months)	NO^b^
Allaix et al.^35^	2015	USA	13	65	Laparoscopy	13	13	24	5
Altorki et al.^18^	1993	USA	17	65	Open	17	17	84	5
Bagheri et al.^23^	2014	Iran	17	39	Open	12	0	12	5
Benacci et al.^8^	1993	USA	33	65	Open	23	6	83	6
Bowman et al.^11^	2015	USA	44	70	Laparoscopy	44	44	39	6
Castrucci et al.^21^	1998	Italy	27	55	Open	22	17	47	4
DJourno et al.^20^	2009	Canada	23	58	Open	23	22	61	5
Fekete and Vonns^10^	1992	France	27	63	Open	15	14	6	5
Fumagalli et al.^33^	2012	Italy	30	62	Laparoscopy	30	30	52	5
Gonzalez-Calatayud et al.^12^	2014	Spain	6	64	Laparoscopy^c^	6	6	62	5
Hauge et al.^13^	2014	Norway	11	60	Both	3	3	27	6
Hudspeth et al.^24^	1993	USA	9	62	Open	6	0	36	5
Jordan and Kinner^25^	1999	USA	19	59	Open	13	4	–	4
Klaus et al.^6^	2003	USA	11	68	Laparoscopy^c^	10	10	26	5
Macke et al.^26^	2015	USA	57	71	Laparoscopy^c^	47	24	21	6
Matthews et al.^27^	2003	USA	5	64	Laparoscopy^c^	5	4	16	6
Melman et al.^32^	2009	USA	13	67	Laparoscopy	13	12	14	6
Nehra et al.^7^	2002	USA	18	66	Open	17	17	24	5
Rossetti et al.^30^	2013	Italy	21	59	Laparoscopy	21	21	78	6
Soares et al.^29^	2011	USA	23	57	Both	21	23	34	6
Streitz et al.^22^	1992	USA	16	62	Open	13	0	84	5
Tedesco et al.^34^	2005	USA	7	73	Laparoscopy	7	7	60	4
van der Peet et al.^28^	2001	Netherlands	5	58	Laparoscopy^c^	2	0	–	4
Varghese et al.^19^	2007	USA	35	71	Open	33	34	45	6
Zaninotto et al.^31^	2012	Italy	24	61	Laparoscopy	21	24	96	5

^a^Median follow-up

^b^Newcastle-Ottawa score

^c^Studies which also utilised thoracoscopy

### Patient Demographics and Diagnosis

Analysis was carried out on 511 patients [259 male, median age (range) 62 years (16–96) with mid-oesophageal (*n* = 53) and epiphrenic (*n* = 458) OD]. The median size (range) of diverticulum was 5 (1–16) cm. Preoperative manometry was performed in 408 patients (79.8%), and oesophageal motility disorders were identified in 363 patients (71%).

### Indications for Surgery

Only one study advocated surgery in asymptomatic patients with non-Zenker’s OD.^18^ Dysphagia and regurgitation were reported in 416 (81.4%) and 365 (71.4%) patients, respectively. Respiratory symptoms of cough and aspiration were reported in 129 (25.2%) patients.

### Surgical Approach

Eleven studies reported outcomes of open surgical approach^7^^,^ 8 ^,^ 10 ^,^ 18 ^–^ 25 in 257 patients (51.6%) [left thoracotomy (*n* = 186), right thoracotomy (*n* = 66) and laparotomy (*n* = 5)]. Seven studies utilised the thoracoscopic approach^6^^,^ 12 ^,^ 13 ^,^ 26 ^–^ 29 in 42 patients. Seven studies utilised laparoscopy alone^11^^,^ 30 ^–^ 35 in 204 patients. Three studies utilised a combined laparoscopic and thoracoscopic approach^12^^,^ 26 ^,^ 28 in eight patients. Nine patients (3.7%) required conversion to open procedure [thoracoscopy to thoracotomy (*n* = 6), laparoscopy to thoracotomy (*n* = 1), laparoscopy to laparotomy (*n* = 1)].

### Management of Diverticulum

Thirteen studies reported outcomes of routine diverticulectomy.^11^^,^ 12 ^,^ 22 ^,^ 24 ^,^ 26 ^–^ 34 Diverticulectomy and diverticulopexy were performed in 456 (89.2%) and 17 (3.3%) patients, respectively. The diverticulum was left in situ in 38 patients (7.4%) who underwent myotomy with or without an anti-reflux procedure.

### Myotomy

Myotomy was performed in 437 (23 mid and 414 distal OD) patients (85.5%). Selective and routine approaches to myotomy were adopted in 15^6^^–^ 8 ^,^ 10 ^,^ 13 ^,^ 19 ^,^ 21 ^–^ 26 ^,^ 28 ^,^ 29 ^,^ 31 and 10^11^^,^ 12 ^,^ 18 ^,^ 20 ^,^ 27 ^,^ 30 ^,^ 32 ^–^ 35 studies, respectively. Myotomy was performed on the contralateral and ipsilateral sides to the diverticulectomy in 11 studies^7^^,^ 10 ^,^ 18 ^–^ 21 ^,^ 23 ^,^ 24 ^,^ 27 ^,^ 32 ^,^ 33 (*n* = 237) and 1 study,^30^ respectively (*n* = 21) and on either side in 1 study^26^ (*n* = 47) and anteriorly in 12 studies^6^^,^ 8 ^,^ 11 ^–^ 13 ^,^ 22 ^,^ 25 ^,^ 28 ^,^ 29 ^,^ 31 ^,^ 34 ^,^ 35 (*n* = 132).

### Fundoplication

Fundoplication was performed in 355 patients (69.5%) [Dor (*n* = 148), Belsey Mark IV (*n* = 100), Toupet (*n* = 63), Nissen (*n* = 44)]. Four studies did not report the use of fundoplication.^22^^–^ 24 ^,^ 28

### Outcomes

#### Staple Line Leak

**Fig. 2:**
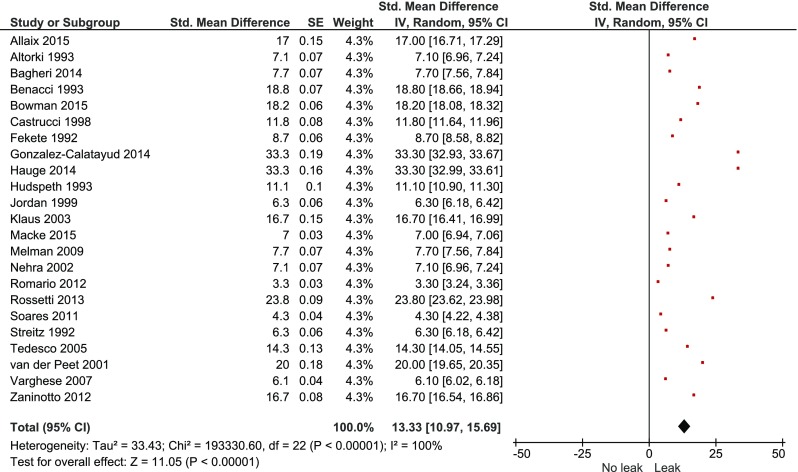
Overall pooled staple line leak rate

Individual study outcomes are shown in Table 2. One study did not report long-term outcomes following surgery.^28^ Staple line leaks were diagnosed either at contrast study or endoscopy in all papers. Staple line leaks occurred in 51 patients, 8 of whom had died. Sixteen patients were treated conservatively with antibiotics and parenteral nutrition, 17 required percutaneous drainage, 15 returned to theatre and 3 patients were stented successfully. Twenty-three studies^6^^–^ 8 ^,^ 10 ^–^ 13 ^,^ 18 ^,^ 19 ^,^ 21 ^–^ 26 ^,^ 28 ^–^ 36 reported at least one staple line leak and were included in the overall pooled estimated leak rate of 13.3% [95% c.i. (11.0–15.7), *p* < 0.001] (Fig. 2). Pooled staple line leak rates according to surgical approach are shown in Table 3.

**Table 2:** Outcomes of individual studies

Author	Total	Diverticulectomy	Leak	Morbidity	Reoperation	Mortality	Recurrence
Allaix et al.^35^	13	6 (46.2)	1 (15.7)	1 (7.7)	0	0	0
Altorki et al.^18^	17	14 (82.4)	1 (7.1)	1 (5.9)	0	1 (5.9)	0
Bagheri et al.^23^	17	13 (76.5)	1 (7.7)	3 (17.6)	0	0	0
Benacci et al.^8^	33	32 (97.0)	6 (18.8)	11 (33.3)	2 (6.1)	3 (9.1)	0
Bowman et al.^11^	44	44 (100.0)	8 (18.2)	33 (75.0)	0	0	0
Castrucci et al.^21^	27	17 (63.0)	2 (11.8)	3 (11.1)	2 (7.4)	2 (7.4)	0
DJourno et al.^20^	23	13 (56.5)	0	2 (8.7)	0	0	0
Fekete and Vonns^10^	27	23 (85.2)	2 (8.7)	5 (18.5)	1 (3.7)	3 (11.1)	2 (7.4)
Fumagalli et al.^33^	30	30 (100.0)	1 (3.3)	2 (6.7)	1 (3.3)	0	0
Gonzalez-Calatayud et al.^12^	6	6 (100.0)	2 (33.3)	2 (33.3)	0	0	0
Hauge et al.^13^	11	9 (81.2)	3 (33.3)	3 (27.3)	2 (18.2)	0	0
Hudspeth et al.^24^	9	9 (100.0)	1 (11.1)	1 (11.1)	1 (11.1)	0	0
Jordan and Kinner^25^	19	16 (84.2)	1 (6.3)	1 (5.3)	0	0	0
Klaus et al.^6^	11	6 (54.5)	1 (16.7)	2 (18.2)	1 (9.1)	0	0
Macke et al.^26^	57	57 (100.0)	4 (7.0)	18 (31.6)	4 (7.0)	1 (1.8)	0
Matthews et al.^27^	5	5 (100.0)	0	0	0	0	0
Melman et al.^32^	13	13 (100.0)	1 (7.7)	2 (15.4)	1 (7.7)	0	0
Nehra et al.^7^	18	14 (77.8)	1 (7.1)	3 (16.7)	2 (11.1)	1 (9.1)	0
Rossetti et al.^30^	21	21 (100.0)	5 (23.8)	6 (28.6)	0	1 (4.8)	0
Soares et al.^29^	23	23 (100.0)	1 (4.3)	5 (21.7)	1 (4.3)	1 (4.3)	0
Streitz et al.^22^	16	16 (100.0)	1 (6.3)	6 (37.5)	0	0	0
Tedesco et al.^34^	7	7 (100.0)	1 (14.3)	1 (14.3)	1 (14.3)	0	0
van der Peet et al.^28^	5	5 (100.0)	1 (20.0)	1 (20.0)	1 (20.0)	0	1 (20.0)
Varghese et al.^19^	35	33 (94.3)	2 (6.1)	5 (14.3)	1 (2.9)	1 (2.9)	0
Zaninotto et al.^31^	24	24 (100.0)	4 (16.7)	6 (25.0)	0	0	0

Percentages in parentheses

**Table 3:** Pooled staple line leak rates according to surgical approach

Surgical approach	Pooled staple line leak rates, (95% c.i.)	*p* value
Open	11.3 (8.4–14.2)	0.347
Minimally invasive	15.2 (11.4–19.0)	
Myotomy	12.4 (9.2–15.6)	0.002
No myotomy	26.1 (18.3–33.9)	
Anti-reflux	14.7 (10.8–18.5)	0.45
No anti-reflux	13.3 (9.9–16.7)	

#### Treatment Success

**Fig. 3:**
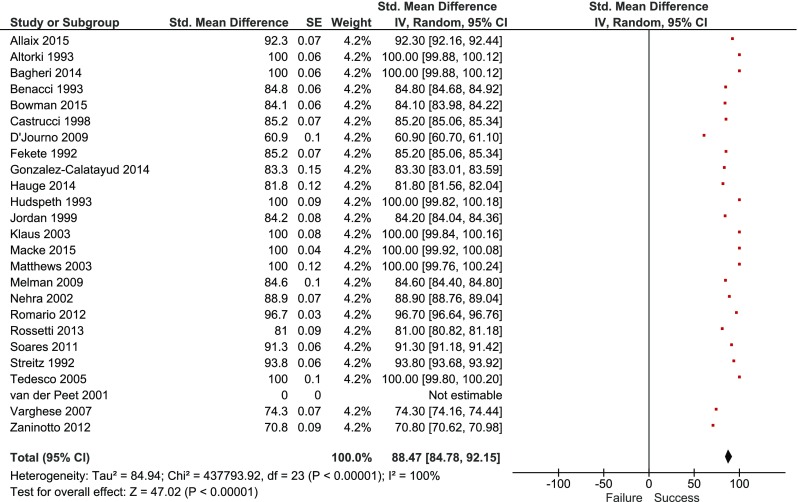
Overall pooled treatment success rate

The overall pooled estimated treatment success rate was 88.5% [95% c.i. (84.8–92.2), *p* < 0.001] (Fig. 3). The treatment success rates according to surgical approach are shown in Table 4.

**Table 4:** Pooled treatment success rates according to surgical approach

Surgical approach	Pooled treatment success rates (95% c.i.)	*p* value
Open	87.4 (81.8–93.0)	0.56
Minimally invasive	89.6 (84.6–94.5)	
Diverticulectomy	85.0 (80.9–89.1)	0.02
No diverticulectomy	65.4 (55.6–75.2)	

#### Morbidity

**Fig. 4:**
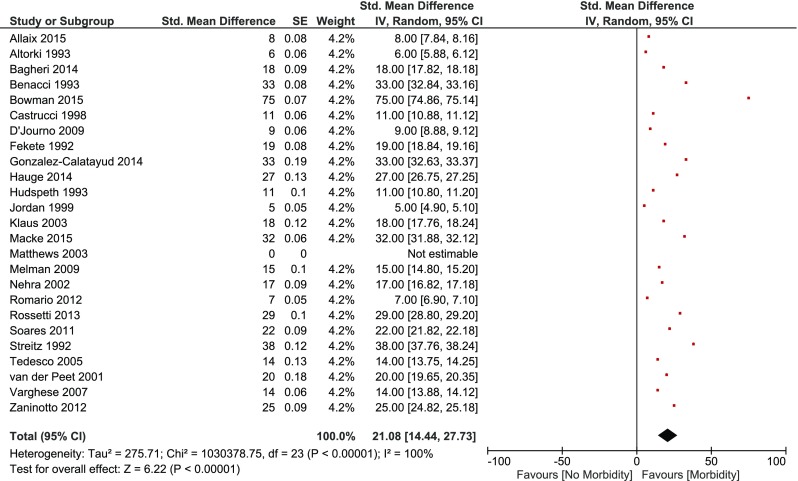
Overall pooled morbidity rate

Morbidity was reported in 111 patients (staple line leak = 51, wound infection = 3, cardiovascular = 17, respiratory = 27, urinary tract infection = 3, bleeding = 3 and “other” = 7). Twenty-four studies^6^^–^ 8 ^,^ 10 ^–^ 13 ^,^ 18 ^–^ 26 ^,^ 28 ^–^ 36 reported at least one complication and were included in the overall pooled estimated morbidity rate of 21.1% [95% c.i. (14.4–27.7), *p* < 0.001] (Fig. 4). Morbidity of open vs. minimally invasive approaches was 17.3% [95% c.i. (12.1–22.5)] and 25.7% [95% c.i. (12.1–39.3), *p* = 0.145], respectively.

#### Reoperation

Twenty patients required reoperations for staple line leak (*n* = 15), bleeding (*n* = 3), port site hernia (*n* = 1), acute paraoesophageal hernia (*n* = 1) and splenic injury requiring splenectomy (*n* = 1). Thirteen^6^^–^ 8 ^,^ 10 ^,^ 13 ^,^ 19 ^,^ 24 ^,^ 26 ^,^ 28 ^,^ 29 ^,^ 32 ^–^ 34 studies reported at least one reoperation and were included in the overall pooled estimated reoperation rate of 9.4% [95% c.i. (7.7–11.1), *p* < 0.001].

#### In-Hospital Mortality

Fourteen patients died in hospital following surgery due to staple line leak (*n* = 8), pneumonia (*n* = 2), myocardial infarction (*n* = 3) and port site hernia (*n* = 1). These were reported in nine studies^7^^,^ 8 ^,^ 10 ^,^ 18 ^,^ 19 ^,^ 21 ^,^ 26 ^,^ 29 ^,^ 30 which were included in the overall pooled estimated in-hospital mortality rate of 5.9% [95% c.i. (4.0–7.8), *p* < 0.001].

#### Recurrence and Reflux

At a median follow-up of 46 months, three patients developed recurrence of the ED which required reoperation, one of whom did not have a myotomy at the index procedure. Postoperative reflux was assessed with routine 24-h pH monitoring in five studies.^20^^,^ 21 ^,^ 29 ^–^ 31 Two studies utilised quantitative assessment of reflux with a modified Likert score,^11^^,^ 34 and one study used the GERD-HRQOL questionnaire.^26^ The rest of the studies assessed postoperative reflux symptoms by simple questioning.

Twelve studies^6^^–^ 8 ^,^ 10 ^,^ 13 ^,^ 18 ^,^ 20 ^–^ 22 ^,^ 29 ^,^ 32 ^,^ 35 reported reflux symptoms at follow-up and were included in the pooled estimated incidence of reflux symptoms which was similarly irrespective of whether an anti-reflux procedure was performed [19.0 (95% c.i. 7.1–30.9%)] or not [21.0 (95% c.i. 13.1–28.9%), *p* = 0.243].

### Sensitivity Analysis and Heterogeneity

Sensitivity analysis of higher quality studies with at least 10 patients revealed a similar pooled staple line leak rate of 14.9% [95% c.i. (10.2–19.6), *p* < 0.001]. Heterogeneity was significant in all analyses.

## Discussion

### Main Findings

The main findings from this meta-analysis of 25 studies of over 500 patients with non-Zenker’s OD were that diverticulectomy resulted in better symptom resolution, and staple line leak rates can be reduced significantly by routine myotomy. Both open and minimally invasive approaches resulted in similar outcomes, and the addition of anti-reflux procedures did not significantly improve postoperative reflux symptoms.

### Strengths

The strengths of this study are the large sample size analysed. Due to the rarity of non-Zenker’s OD, the controversies surrounding the surgical treatment of these patients will not be answered by randomised trials. This is the only comprehensive meta-analysis of the outcomes of surgery in over 500 patients with non-Zenker’s OD which has identified the optimum treatment. The largest case series to date only included 57 patients over a 15-year period.^26^ A nationwide population database of 1056 patients with non-Zenker’s OD reported a leak rate of 3.1%^37^ which is at odds with the findings of our study. Hospital coding was used in this database which may have underestimated the complication rates. These types of studies were therefore not included in our meta-analysis.

### Limitations

This study has limitations. Meta-analysis of retrospective cohort studies is regrettably sensitive to confounding and selection bias. However, there are no randomised trials comparing the various surgical approaches. A variety of procedures were used in the studies included in the meta-analysis resulting in significant heterogeneity. The outcomes (staple line leakage and success rates) were not explicitly defined in all papers and not stratified according to the site of the diverticula. The assessment of symptoms at follow-up also varied significantly between studies. We therefore broadly defined success rates as symptom improvement or resolution at follow-up which was reported in all studies. Subgroup analysis was limited as not all studies reported separate outcomes according to the presence of motility disorders or individual surgical approach. Nevertheless, a sensitivity analysis of higher quality studies revealed similar results to the overall analysis, thereby strengthening the conclusions.

### Surgical Approach

Despite the increased use of minimally invasive approaches since 2000, the open approach is still widely adopted. Over half of patients in this cohort underwent open surgery usually via a left thoracotomy. Although the treatment success rates were similar between the two approaches, there was a non-significant trend towards higher staple line leak and overall morbidity rates in patients who underwent minimally invasive surgery. Short-term outcomes, for example length of hospital stay, appear to be shorter in individual series reporting the minimally invasive approach,^6^^,^ 29 ^,^ 34 but this could not be analysed as only less than half of the studies included in this meta-analysis reported length of hospital stay. The choice of approach depends not only on the location of the OD, need for myotomy and anti-reflux procedure but, more importantly, on local expertise. Minimally invasive approaches should only be performed by surgeons experienced in both open and minimally invasive oesophageal surgeries.^26^

### Management of Diverticulum

The majority of patients in this study underwent excision of the OD. Castrucci et al. did not perform a diverticulectomy in the presence of wide-necked diverticula without food retention in the pouch, pulmonary aspiration or mucosal lesions.^21^ D’Journo et al. advocated suspension of wide-necked diverticula when there was no dependent portion of the diverticular sac and myotomy alone in the presence of multiple small diverticula.^20^ Small diverticula are usually less symptomatic^6^^,^ 21 and should arguably be treated non-surgically^32^ unless the predominant symptom is dysphagia secondary to achalasia. Diverticulectomy resulted in improvement or resolution in symptoms in 85% of patients compared with 65% who underwent diverticulopexy or myotomy alone. Excision of the OD should therefore be performed in the presence of symptoms directly related to the OD such as food regurgitation.

### Myotomy

Another contentious issue is the need for myotomy. The pathogenesis of non-Zenker’s OD is not fully understood. The diagnosis of oesophageal motility disorders is challenging and the current method of investigation is not tolerated by all patients. Some studies have identified motility disorders in almost all patients with non-Zenker’s OD^7^^,^ 11 ^,^ 30 ^,^ 35 whereas others have identified motor disorders in less than 20%.^6^^,^ 13 These differences between series may be explained by a variation in criteria used to reach a diagnosis^38^ or the intermittent dysfunction that is not detected by oesophageal motility studies.^7^ Oesophageal motor disorders were identified in just over 70% of patients, and myotomy was performed in 85% of patients in this meta-analysis. Just as Belsey^5^ pointed out over half a century ago, the underlying cause leading to the blow out must be addressed if successful surgery is expected. We have shown that myotomy significantly reduces the staple line leak rate from 26 to 12.4%.

### Anti-reflux

The need for an anti-reflux procedure and the type of fundoplication are widely debated topics. Over two thirds of patients in this study underwent an anti-reflux procedure, the majority of whom had a partial fundoplication. The staple line leak rates and postoperative reflux rates were similar regardless of whether a fundoplication was performed or not. However, these results should be interpreted with caution as the reporting of symptomatic reflux outcomes varied between studies. Moreover, it was not possible to identify the optimum type of fundoplication in this meta-analysis as the outcomes of the various procedures were not reported separately. The choice of fundoplication is therefore dependent on the patients’ symptoms and surgeon preference.

## Conclusion

In conclusion, this comprehensive meta-analysis of over 500 patients with non-Zenker’s OD has shown that the optimum surgical treatment is diverticulectomy along with routine myotomy with or without an anti-reflux procedure. Both open and minimally invasive approaches are equally effective.
